# Neurotoxic Methamphetamine Doses Increase LINE-1 Expression in the Neurogenic Zones of the Adult Rat Brain

**DOI:** 10.1038/srep14356

**Published:** 2015-10-14

**Authors:** Anna Moszczynska, Amanda Flack, Ping Qiu, Alysson R. Muotri, Bryan A. Killinger

**Affiliations:** 1 Department of Pharmaceutical Sciences, College of Pharmacy and Health Sciences, Wayne State University, Detroit, MI 48202; 2 Departments of Pediatrics/Rady Children’s Hospital San Diego, Department of Cellular & Molecular Medicine, Stem Cell Program, University of California San Diego, School of Medicine, La Jolla, CA 92093.

## Abstract

Methamphetamine (METH) is a widely abused psychostimulant with the potential to cause neurotoxicity in the striatum and hippocampus. Several epigenetic changes have been described after administration of METH; however, there are no data regarding the effects of METH on the activity of transposable elements in the adult brain. The present study demonstrates that systemic administration of neurotoxic METH doses increases the activity of Long INterspersed Element (LINE-1) in two neurogenic niches in the adult rat brain in a promoter hypomethylation-independent manner. Our study also demonstrates that neurotoxic METH triggers persistent decreases in LINE-1 expression and increases the LINE-1 levels within genomic DNA in the striatum and dentate gyrus of the hippocampus, and that METH triggers LINE-1 retrotransposition *in vitro.* We also present indirect evidence for the involvement of glutamate (GLU) in LINE-1 activation. The results suggest that LINE-1 activation might occur in neurogenic areas in human METH users and might contribute to METH abuse-induced hippocampus-dependent memory deficits and impaired performance on several cognitive tasks mediated by the striatum.

Methamphetamine (METH) is a potent and widely abused central nervous system (CNS) psychostimulant that has been one of the major public health concerns worldwide since the late 1990s. METH abuse causes a broad range of severe cognitive deficits^1^ as well as neurobehavioral abnormalities, such as aggressive and psychotic behavior^2^, which are related to the neurotoxic effects of METH on the CNS. At high doses, METH causes the degeneration of dopaminergic (DAergic) and serotonergic nerve terminals, particularly in the striatum^3^. In neurons that are post-synaptic to striatal monoaminergic terminals, METH causes apoptosis, and cell death in some species^4^,^5^,^6^,^7^,^8^. In the hippocampus, METH dysregulates neurogenesis and induces apoptosis, which is often followed by the death of pyramidal neurons and granular cells^8^,^9^,^10^,^11^,^12^,^13^,^14^. Clinical studies in human METH users have found that the METH-induced long-term deficits in DAergic components in the striatum are correlated with cognitive decline and poor psychomotor functioning^15^, whereas the METH effects on the hippocampus play a role in long-term memory^1^.

Despite years of active research, there are no specific medications that can counteract the damaging effects of METH on adult brain. In recent years, epigenetics has attracted much attention as a novel and promising research area in METH abuse^16^. Most studies have investigated epigenetic changes in the nucleus accumbens that are induced by non-toxic doses of METH and have focused on histone modifications and global or gene-specific DNA methylation^16^,^17^,^18^,^19^,^20^,^21^,^22^,^23^,^24^. Several of these investigations examined amphetamine-induced epigenetic changes in the striatum^17^,^18^,^20^,^21^,^22^ and hippocampus^19^,^23^,^24^,^25^ using a variety of regimens and detected alterations in several epigenetic indices. Only a handful of studies employed neurotoxic doses of acute or chronic METH and found that self-administration of high-dose METH triggered changes in histone modifications and the expression of genes coding for proteins involved in chromatin remodeling^26^,^27^, whereas neurotoxic binge METH decreased the expression of several histone deacetylases (HDACs)^28^ in the striatum. In the substantia nigra, high-dose METH injected over four days decreased DNA methylation within the promoter region of alpha-synuclein^29^.

Chromatin structure (via histone modifications), HDACs, and DNA methylation regulate transposable elements (TEs)^30^,^31^,^32^, which are repetitive DNA sequences that can induce epigenetic alterations in the genome^33^,^34^. There are no data on the effects of METH on TEs *in vivo*. In neuronal cell lines, METH has been shown to trigger retrotransposition of Long INterspersed Element-1 (LINE-1)^35^. LINE-1 is the most abundant and the most active autonomous TE and is highly conserved in human and rodent DNA; it is dormant in most somatic cells and active during neurogenesis^33^,^34^,^36^. Dysregulation of LINE-1 expression or retrotransposition contributes to several neurological diseases and can be triggered by substance abuse^34^. For example, LINE-1 expression in the nucleus accumbens increases after chronic administration of psychostimulant cocaine to mice^30^; the effect is accompanied by a decrease in trimethylated histone 3, a LINE-1-binding protein.

Based on the data from the literature and the fact that TEs do not undergo retrotransposition in non-proliferating cells, we hypothesized that neurotoxic binge METH would increase LINE-1 expression and the genomic DNA (gDNA) copy number in two neurogenic areas in the adult brain; the subgranular zone (SGZ) of the dentate gyrus and the subventricular zone (SVZ), which is located between the lateral ventricle and the striatum (Fig. 1A,B). The LINE-1 element consists of a promoter, 2 open reading frames (ORF-1 and ORF-2) and a polyA tail (Fig. 1C). LINE-1 activation, most often by promoter hypomethylation, leads to ORF-1 and ORF-2 translation in the cytoplasm, which can be followed by LINE-1 insertion into the genome. To test our hypothesis *in vivo*, we measured LINE-1 promoter methylation, ORF-1 messenger RNA (mRNA) levels, ORF-2 protein levels, and ORF-1 gDNA copy number in the rat brain. Cultured PC12 cells were used to elucidate the molecular mechanism mediating the increase in LINE-1 expression. We present evidence that binge METH increases ORF-2 protein levels in the neurogenic zones as well as ORF-1 mRNA levels and ORF-1 copy number within gDNA in the rat dentate gyrus and striatum. We also provide *in vitro* data implicating METH-induced glutamate (GLU) toxicity in LINE-1 activation. These findings add to the knowledge of LINE-1 activity in neurons exposed to severe oxidative stress and suggest that activation of LINE-1 *in vivo* is a consequence of exposure to METH.

## Results

### Binge METH rapidly increases ORF-1 mRNA levels in the striatum and dentate gyrus of the adult rat brain

**Figure 2:**
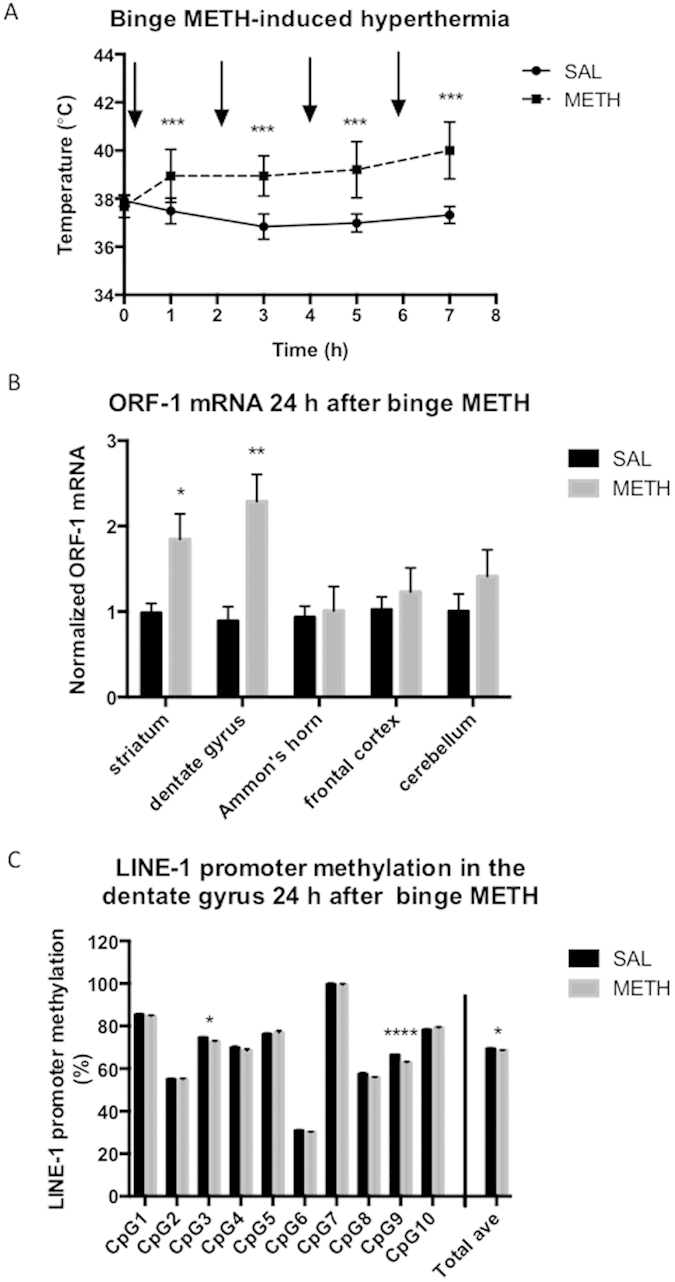
METH-induced hyperthermia and short-term effects of binge METH on the levels of ORF-1 mRNA in the rat brain. Adult male Sprague-Dawley rats were administered saline (1 mL/kg) or binge METH (4 × 10 mg/kg, i.p. every 2 h) and killed 24 h later. (**A**) METH-induced hyperthermia. Core body temperatures (°C) were measured before treatments and 1 h after each METH or saline injection. The black arrows indicate the injection times. Binge METH induced significant hyperthermia during the treatment (***p < 0.001, two-way ANOVA with repeated measures followed by the Student-Newman-Keuls *post hoc* test, n = 4–7/group). (**B**) Short-term effect of binge METH on the levels of ORF-1 mRNA in rat brain. Compared with the controls, METH significantly increased ORF-1 mRNA levels in the striatum (1.8-fold, *p < 0.025) and the dentate gyrus (2.3-fold, **p < 0.01) (Student’s two-tailed *t*-test followed by the Bonferroni correction, n = 4–6 rats/group) at 24 h after METH administration. The data were normalized to the saline controls. (**C**) Short-term effect of binge METH on LINE-1 promoter methylation in the rat brain. The first ten CpG sites within the promoter region of LINE-1 were analyzed for methylation status (%). A small (−1%) but significant (*p* < 0.05, Student’s two-tailed *t*-test, n = 5–7 rats/group) decrease in the average methylation of these sites was observed in the dentate gyrus of binge METH-exposed rats. The decrease was due to the hypomethylation of CpG9 and CpG3 (−3.5%, ****p < 0.0001, and −1.9%, *p < 0.005, respectively, Student’s two-tailed *t*-test followed by the Bonferroni correction for multiple comparisons, n = 5–7 rats/group). The data are expressed as the mean ± SEM. Abbreviations: ave, average; CpG, cytosine guanine dinucleotide; h, hours; METH, methamphetamine; mRNA, messenger ribonucleic acid; SAL, saline.

Severe hyperthermia during METH administration can serve as an indicator of the subsequent neurotoxicity of the drug; therefore, the core body temperature of each rat was recorded before, during, and after the administration of METH. As expected, METH triggered hyperthermia; i.e., METH administration caused significant increases in core body temperatures over time (*p* < 0.001, two-way ANOVA with repeated measures followed by Student-Newman-Keuls *post hoc* test; n = 4–7 rats/group) reaching 40°C after the last METH injection (Fig. 2A).

In the adult brain, LINE-1 retrotransposition occurs in neurogenic areas such as the SGZ, which lies within the dentate gyrus and the SVZ, which is adjacent to the lateral ventricle and striatum. Therefore, we first examined whether binge METH augments LINE-1 ORF-1 mRNA and ORF-2 protein levels in these neurogenic niches in the rat brain. METH significantly increased ORF-1 mRNA levels in the dentate gyrus and striatum at 24 h after the last injection of the drug (by 2.3-fold, *p* < 0.01, *t* = 3.69, *df* = 11, and 1.8-fold, *t* = 3.15, *df* = 8, *p* < 0.025, respectively; Student’s two-tailed *t*-test followed by the Bonferroni correction for multiple comparisons, n = 4–7 rats/group). This result suggests that binge METH augmented LINE-1 transcription in both regions at a point between the beginning of METH administration and 24 h after the last dose of the drug. Administration of cocaine, morphine or alcohol, increases LINE-1 expression in non-neurogenic brain areas^34^,^37^. Hence, we next assessed ORF-1 mRNA levels in Ammon’s horn of the hippocampus (CA1 and CA3), in the frontal cortex, and in the cerebellum. Rats were treated with METH or saline and were sacrificed 24 h after the last injection. METH did not affect the ORF-1 mRNA levels in any of these areas (*p* > 0.1, Student’s two-tailed *t*-test followed by the Bonferroni correction for multiple comparisons, 5 groups, n = 4–8 rats/group). The data are summarized in Fig. 2B.

There was no correlation between dentate gyrus or striatal ORF-1 mRNA levels and hyperthermia (ORF-1 mRNA levels *vs.* area under the temperature curve) (Pearson two-tailed correlation analysis, *r* = −0.0051, *p* = 0.99 and *r* = −0.279, *p* = 0.72, respectively), suggesting that the increase in ORF-1 mRNA levels was not caused by the increase in core body temperature.

### Binge METH increases ORF-2 protein levels in neurogenic zones

**Figure 3:**
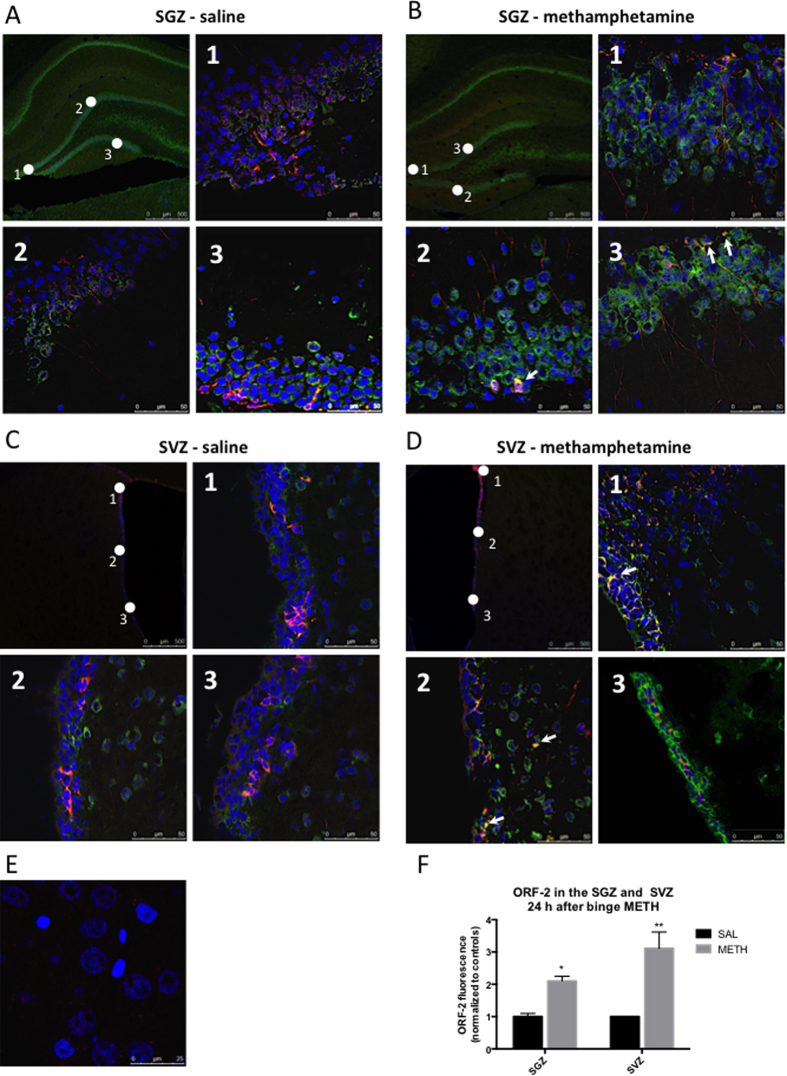
Short-term effects of binge METH on the levels of ORF-2 protein in the (A) subgranular zone (SGZ) of the dentate gyrus and (B) subventricular zone (SVZ). Adult male Sprague-Dawley rats were administered saline (1 mL/kg) or binge METH (4 × 10 mg/kg, i.p. every 2 h) and killed 24 h later. Representative images from 3 regions of the SGZ (**A,B**) and the SVZ (**C,D**) per condition (SAL vs. METH). METH-treated rats displayed higher ORF-2 protein immunoreactivity (green) in the SGZ (B vs. A) and SVZ (D *vs.* C) than the saline controls did (by 2.1-fold, *p < 0.0125, and 3.1-fold, **p < 0.005, respectively, Student’s two-tailed *t*-test with the Bonferroni correction). The data are summarized in (**F**). Low ORF-2 protein immunoreactivity, concentrated in the perinuclear region, was detected in saline-treated rats in both zones (**A,C**). Many, but not all, ORF-2-positive neurons were also positive for doublecortin (arrows) (red), which is a selective marker of cells committed to the neuronal lineage, in both saline- and METH-treated rats. (**E**) Secondary antibody control. Nuclei are depicted in blue. Bars: (**A-D**) 50 μm, (**E**) 25 μm.

LINE-1 is activated and readily retrotransposes in proliferating cells. Consequently, we next examined rat brains for localization of ORF-2 protein immunoreactivity in the SGZ and the SVZ. Low ORF-2 protein immunoreactivity, concentrated in the perinuclear region, was detected in both the SGZ and SVZ zone in saline-treated rats (Fig. 3A, C). ORF-2 immunoreactivity was also detected in the striatum adjacent to the SVZ. At 24 h after the last injection, the METH-treated rats displayed higher ORF-2 protein immunoreactivity in the SGZ (by 2.1-fold, *p* < 0.005, Student’s two-tailed *t*-test with the Bonferroni correction, *t* = 6.30, *df* = 4) and in the adjacent granular cell layers than the saline-treated controls (Fig. 3B). A similar effect of METH was observed in the SVZ; binge METH-treated rats expressed more ORF2 protein in the SVZ than did the saline controls (by 3.1-fold, *p* < 0.025, Student’s two-tailed *t*-test with the Bonferroni correction, *t* = 4.21, *df* = 4) (Fig. 3C). In addition, an increased ORF-2 signal was observed outside the SVZ, in the portion of the striatum adjacent to the SVZ (Fig. 3D). Many, but not all, ORF-2-positive neurons were also positive for doublecortin, a selective marker of cells committed to the neuronal lineage, in both saline- and METH-treated rats.

### Binge METH-triggered activation of LINE-1 is accompanied by low-level LINE-1 promoter hypomethylation in the dentate gyrus

LINE-1 activation is often induced by hypomethylation of its promoter region^31^,^38^. Examination of the first ten CpG sites within the promoter of LINE-1 revealed a small (−1%) but significant (*p* < 0.025, Student’s one-tailed *t*-test with the Bonferroni correction for two comparisons (dentate gyrus, striatum), n = 5–7 rats/group) decrease in the average methylation of these sites in the dentate gyrus of binge METH-exposed rats relative to saline controls at the 24 h time point (Table 1). As subsequently determined, the decrease was due to hypomethylation of CpG9 and CpG3 (−3.5%, *p* < 0.0001, *t* = 7.84, *df* = 10 and −1.9%, *p* < 0.005, *t* = 4.47, *df* = 10, respectively, Student’s one-tailed *t*-test followed by the Bonferroni correction for multiple comparisons of CpGs, n = 5–7 rats/group) (Fig. 2C). Pearson’s correlation analysis did not reveal significant correlations between ORF-1 mRNA levels and the average methylation of CpGs 1–10 (Pearson’s *r* = 0.188, *p* = 0.685, n = 7 rats/group), or between ORF-1 mRNA levels and the methylation of CpG9 (Pearson’s *r* = 0.291, *p* = 0.526, n = 7 rats/group), or between ORF-1 mRNA levels and the methylation of CpG3 (Pearson’s *r* = 0.235 *p* = 0.511, n = 7 rats/group). In the striatum, no significant hypomethylation was detected in METH-treated rats compared with the saline controls. Examination of LINE-1 promoter hypomethylation in the remaining hippocampus, frontal cortex, and cerebellum also did not reveal any significant changes. To determine whether LINE-1 promoter hypomethylation in the dentate gyrus and striatum occurred at an earlier time point than 24 h after the last METH dose, the rats were treated with binge METH or saline and sacrificed at 1 h after the last injection. No significant changes in LINE-1 promoter methylation were detected in the dentate gyrus or striatum (Table 2). These findings suggest that hypomethylation might not be a major factor in LINE-1 activation after the administration of binge METH.

### Binge METH induces a persistent increase in the ORF-1 gDNA levels in the striatum and dentate gyrus of the adult rat brain

**Figure 4:**
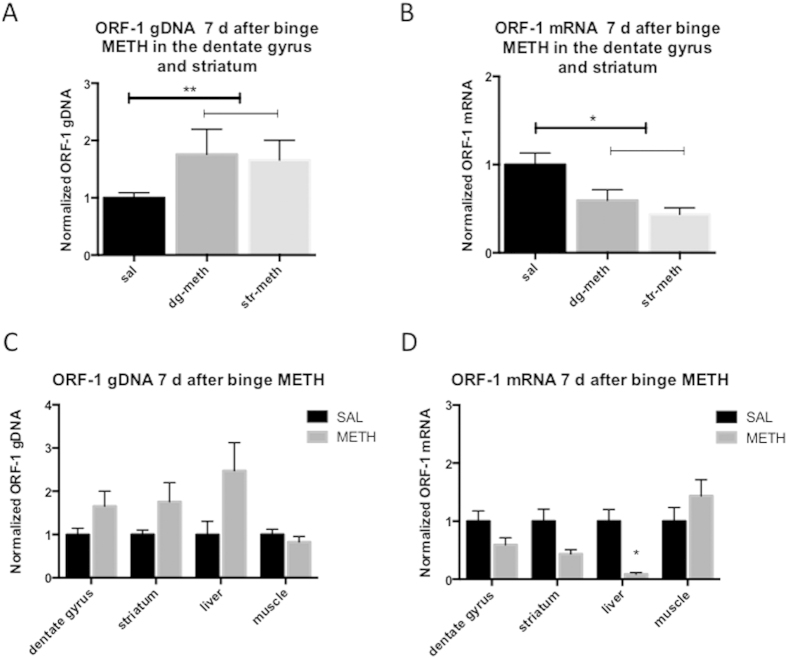
Long-term effect of binge METH on the levels of ORF-1 mRNA and gDNA in rat brain, liver and muscle. Adult male Sprague-Dawley rats were administered saline (1 mL/kg) or binge METH (4 × 10 mg/kg, i.p. every 2 h) and sacrificed 7 days after the treatment. Compared with the saline controls, METH significantly increased ORF-1 gDNA levels **(A**) and decreased ORF-1 mRNA levels (**B**) in the neurogenic regions of the brain (dentate gyrus and striatum combined) (+71%, **p < 0.01, n = 8–11 and −49%, *p < 0.05, n = 5–7, respectively, Student’s two-tailed *t*-test). (**C**) When ORF-1 gDNA was assessed in the striatum, dentate gyrus, liver, and muscle, no significant changes were detected using the Student’s *t*-test with the Bonferroni correction. Data unadjusted for multiple comparisons revealed an increase in ORF-1 gRNA in the dentate gyrus and striatum and a statistical trend for an increase in the liver (+66%, *p* < 0.05, +76%, *p* < 0.05 and 2.5-fold, *p* = 0.064, respectively, Student’s one-tailed *t*-test). (**D**) When ORF-1 mRNA was assessed in the striatum, dentate gyrus, liver, and muscle (using Student’s *t*-test with the Bonferroni correction), a significant decrease was observed only in the liver (−91%, *p < 0.0125). Data unadjusted for multiple comparisons revealed a decrease in ORF-1 mRNA in the dentate gyrus and striatum (-41%, *p* < 0.05 and -57%, *p* < 0.05, respectively, Student’s one-tailed *t*-test). The data are expressed as the mean ± SEM. All data were normalized to the saline controls. Abbreviations: d, days; dg, dentate gyrus; gDNA, genomic deoxyribonucleic acid; METH, methamphetamine; mRNA, messenger ribonucleic acid; str, striatum; SAL, saline.

LINE-1 undergoes retrotransposition via a copy-and-paste mechanism and thereby increases its copy number within gDNA^37^. We next determined whether binge METH causes persistent increases in the levels of ORF-1 gDNA in the striatum and dentate gyrus. When the dentate gyrus and striatum were combined into one neurogenic group, the ORF-1gDNA levels were significantly increased (+71%, *p* < 0.01, Student’s two-tailed *t*-test, *t* = 13.7, *df* = 2, n = 8–11 rats/group) in METH-treated rats compared with the saline controls (Fig. 4A), suggesting potential LINE-1 retrotransposition in the SGZ and SVZ. Examination of liver and muscle tissue revealed an increase in the levels of ORF-1 copy numbers in the liver (2.5-fold), but not in the muscle tissue, relative to the saline controls (Fig. 4C). Analysis of the data by Student’s *t*-test with the Bonferroni correction did not reveal significant differences between the saline and METH groups.

### Binge METH regimen leads to a persistent decrease in ORF-1 mRNA levels in the dentate gyrus of the adult rat brain

Binge METH-induced neurotoxicity develops over 3–5 days^3^. To determine whether ORF-1 mRNA levels remain increased in the striatum and dentate gyrus after METH-induced neurodegeneration has occurred, METH-treated and control adult rat brains were examined for the levels of ORF-1 mRNA at 7 days after binge METH treatment. When the dentate gyrus and striatum were combined into one neurogenic group, ORF-1 mRNA levels were significantly decreased (−49%, *p* < 0.05, Student’s two-tailed *t*-test, *t* = 6.1, *df* = 2, n = 5–7 rats/group) in METH-treated rats compared with the saline controls (Fig. 4B). As presented in Fig. 3D, the ORF-1 mRNA levels showed a significant decrease only in the liver when the data were analyzed with Student’s *t*-test with the Bonferroni correction (−91%, *p* < 0.0125). The levels of ORF-1 mRNA in muscle tissue did not significantly differ between METH- and saline-treated rats (Fig. 4D). The results suggested that binge METH-induced LINE-1 activation was followed by an adaptive decrease in LINE-1 expression. Interestingly, the ORF-1 mRNA levels negatively correlated with the ORF-1 gDNA levels in the dentate gyrus (*r* = −0.744, *p* < 0.05, n = 6, Pearson’s analysis) but not in the striatum. The power of the analysis was π = 0.55; nevertheless, the correlation suggests that the reduction in ORF-1 mRNA levels in the dentate gyrus might be partially due to LINE-1 translocation from the cytoplasm to the nucleus.

### METH triggers GLU-mediated LINE-1 retrotransposition in neuronal cells

**Figure 5:**
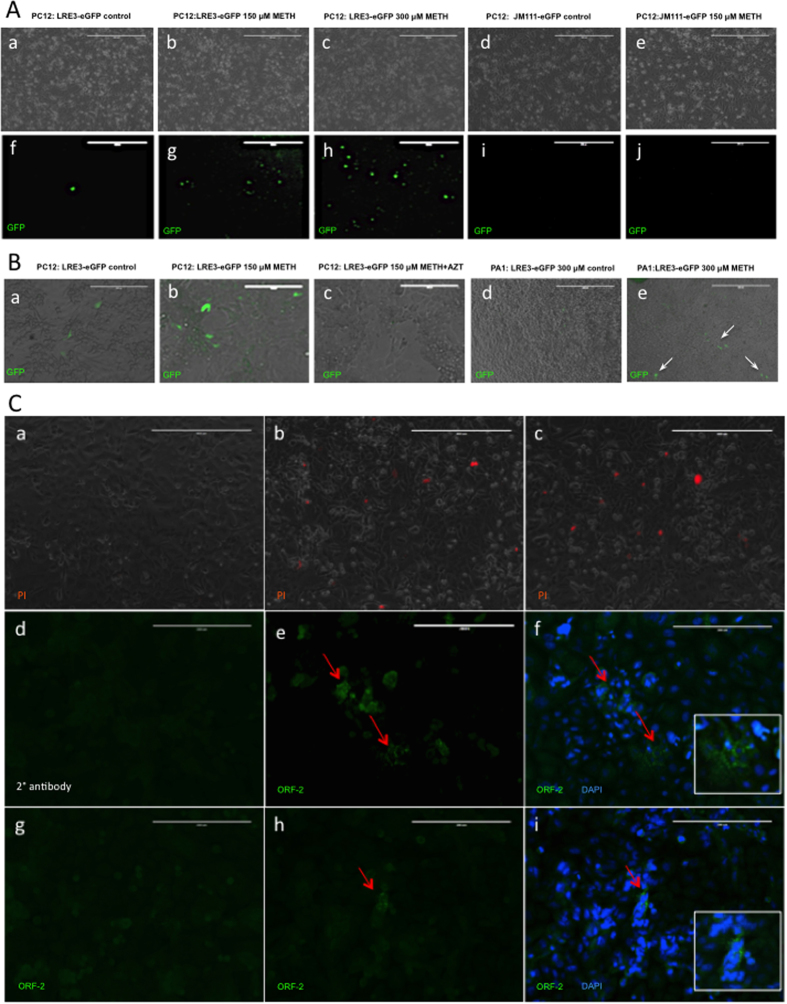
Effect of METH on LINE-1 retrotransposition in dopaminergic neuronal PC12 cells and non-dopaminergic non-neuronal PA-1 cells. Cells were transfected with either an active LINE-1 (LRE3-eGFP) or retrotransposition-defective LINE-1 (JM111-eGFP). (**A**) In untreated PC12 cells, eGFP expression (green) was detected after 10–14 days in a very few cells (**f**). PC12 cells transfected with JM111-eGFP did not show eGFP immunofluorescence (**i,j**). Incubation of LRE3-eGFP-transfected PC12 cells with 0.150 mM or 0.300 mM METH for 10–14 days resulted in higher LINE-1 retrotransposition as manifested by stronger eGFP fluorescence (**g,h**). (**B**) Incubation of PA-1 cells with 0.300 mM METH also resulted in the appearance of an eGFP signal (white arrows) at a higher METH concentration; the eGFP signal was of lower intensity than the eGFP signal in PC12 cells (Be *vs.* Ah). Incubation of LRE3-eGFP-transfected PC12 cells with a LINE-1 retrotransposition inhibitor, azidothymidine (AZT), completely abolished the eGFP signal (c vs. a,b). No eGFP signal was detected in untreated PA-1 cells (**d**). (**C**) Administration of 2 mM glutamate (GLU) or dopamine (DA) for 4 h decreased PC12 cell viability as evidenced by increased propidium iodide (red) accumulation (a, no treatment; b, DA; c, GLU). Compared with untreated PC12 cells (Cg), the 2 mM GLU treatment induced a greater increase in ORF-2 immunoreactivity (green, red arrows) (Ce,f) than 2 mM DA treatment did (Ch,i). (Dg) Secondary antibody negative control. Inserts present magnifications of red arrow-marked areas containing ORF-2 immunoreactivity. Abbreviations: eGFP, enhanced green fluorescent protein; METH, methamphetamine; SAL, saline. Bars: (A,Ca-c) 400 μm, (B,Cd-i) 200 μm.

Exposure of neuronal DAergic PC12 cells to millimolar concentrations of METH *in vitro* triggers DA-mediated neurotoxic events, including apoptosis and oxidative DNA damage^39^. LINE-1 undergoes retrotransposition in PC12 cells after 3 days of exposure to 0.5 mM METH^35^. To test whether LINE-1 undergoes retrotransposition in PC12 cells and in PA-1 cells (which are non-neuronal) after METH treatment, the cells were exposed to 0.150 and/or 0.300 mM of the drug for 10–14 days after transfection with constructs containing a LINE-1 retrotransposition indicator cassette (LRE3-eGFP) or retrotransposition-defective LINE-1 (containing two missense mutations in ORF1) (JM111-eGFP) as well as the puromycin resistance gene^36^. Cells harboring the constructs were selected by the addition of puromycin to the culture medium and screened for eGFP fluorescence. In PC12 cells, the GFP signal appeared, in a dose-dependent manner, after 10–14 days of METH treatment (Fig. 5A,a–c,f–h). Cells transfected with retrotransposition-defective LINE-1 did not show eGFP immunofluorescence (Fig. 5A,d-e,i–j). To determine whether METH is able to trigger LINE-1 retrotransposition in non-neuronal cells (specifically, ovarian cancer cells), PA-1 cells were exposed to 0.300 mM METH while PC12 cells were exposed to 0.150 mM METH. As previously, the incubation of PC12 cells harboring retrotransposition-capable LINE-1 with 0.150 mM METH triggered LINE-1 retrotransposition (Fig. 5B,a–b). Exposure of PC12 cells to METH and a LINE-1 retrotransposition inhibitor (azidothymidine, AZT) decreased the number of GFP-positive cells (Fig. 5B,c), thus confirming that LINE-1 retrotransposition was the source of the eGFP signal. Incubation of PA-1 cells with 0.300 mM METH also resulted in generation of eGFP fluorescence, but the signal was weaker than in PC12 cells (Fig. 5B,d-e). These findings indicate that METH can induce LINE-1 retrotransposition in neuronal and non-neuronal cells. The neurotoxic effects of METH on postsynaptic neurons are mediated via increased neurotransmission of DA and GLU^3^. To determine whether DA and/or GLU mediate an increase in LINE-1 expression, PC12 cells were treated with 2 mM DA or 2 mM GLU for 4 h. Both neurotransmitters decreased cell viability as assessed with propidium iodide (Fig. 5C,a–c). Compared with untreated cells, GLU increased ORF-2 immunoreactivity more than DA did (Fig. 5c,e–i). ORF-2 immunoreactivity was not detected in JM111-eGFP-transfected cells (not shown).

## Discussion

The present study demonstrates that the systemic administration of binge METH, at neurotoxic doses, to adult male rats increases the ORF-1 mRNA and ORF-2 protein levels in the SGZ and SVZ at 24 h after the last dose of the drug, in a LINE-1 promoter hypomethylation-independent manner. Our study also demonstrates that binge METH triggers persistent (up to 7 days after METH regimen) decreases in ORF-1 mRNA levels and increases the levels of ORF-1 gDNA in the dentate gyrus and striatum. The *in vitro* component of the investigation presents evidence for METH-triggered LINE-1 retrotransposition in neuronal and non-neuronal cells and implicates the neurotransmitter GLU in the increases in LINE-1 expression.

Binge METH significantly increased ORF-1 mRNA levels in the dentate gyrus and striatum at 24 h after binge METH administration. Increased LINE-1 expression at the 24 h time point has also been found in the mouse nucleus accumbens after exposure to another psychostimulant, cocaine^30^, and in cultured DAergic cells exposed to morphine^40^, as well as in several brain areas of alcoholics^41^, suggesting a common pathway of LINE-1 induction by these substances. The morphine-induced increase in LINE-1 expression was found to be triggered by the inhibition of cysteine transport into SH-SY5Y cells and the consequent deficit in intracellular GSH^40^. GSH deficit can be generated by severe oxidative stress (mediated by DA autoxidation or mitochondrial dysfunction) or by exposure to GLU, which inhibits cysteine transport^42^. In fact, binge METH induces oxidative stress, mitochondrial impairment, and a GSH deficit in the rodent striatum^4^,^5^,^6^,^7^,^43^,^44^ and hippocampus^8^,^43^,^45^,^46^,^47^, as well as triggers DA and GLU release in these areas^48^,^49^. Oxidative stress, mitochondrial impairment, and a GSH deficit have all been demonstrated to increase LINE-1 mRNA levels in culture^50^,^51^. METH, cocaine, morphine, and alcohol all can induce oxidative stress and a deficit in GSH^40^,^44^,^52^, suggesting that an imbalanced in redox status is a common final pathway leading to LINE-1 activation, with GLU release, rather than DA release, mediating the imbalance. The notion of GLU as major mediator of LINE-1 overproduction is supported by our finding of increased ORF-2 immunoreactivity in PC12 cells treated with GLU (but not in cells treated with DA) and by a report of attenuated METH-induced cell death in the dentate gyrus by the inhibition of GLU release^46^.

The investigation into sites of LINE-1 activation revealed significantly increased levels of ORF-2 protein in the SGZ and SVZ, indicating that METH induces LINE-1 translation mainly in neurogenic areas. Moreover, METH increased the levels of ORF-2 protein in the portion of the striatum adjacent to the SVZ, which might have been a result of local neurogenesis^53^, migration of precursor cells generated in the SVZ to the damaged striatum^54^, or increased LINE-1 expression in a sub-population of striatal cell bodies. ORF-2 immunostaining was present in both doublecortin-positive and doublecortin-negative cells, indicating increased LINE-1 activation in neuronal precursors differentiating into neurons (in agreement with the results of Muotri and colleagues^36^), as well in other cell types. *In vitro*, we detected METH-induced LINE-1 retrotransposition in neuronal PC12 cells, which agrees with a previous study^35^, as well as in non-neuronal PA-1 cells, albeit to a lesser extent, which supports our *in vivo* findings of increased LINE-1 activity in doublecortin-negative cells. In terms of the involvement of GLU in mediating the increases in LINE-1 expression, METH may increase extracellular GLU levels within neurogenic areas via DA release from DAergic terminals followed by GLU release from astrocytes^55^. The SGZ and SVZ both contain the neurotransmitters GLU and DA^56^. Binge METH-induced neurodegeneration takes at least 3 days^3^; therefore, the changes in LINE-1 expression were not related to METH withdrawal. It remains to be determined whether the observed changes promote METH neurotoxicity, which is a likely scenario because a transient increase in LINE-1 expression, particularly in ORF-2, is cytotoxic^57^,^58^.

High doses of METH induce DNA breaks^8^,^59^ and apoptotic and necrotic death of SGZ and SVZ cells^9^,^46^,^60^. LINE-1 can “jump” into DNA at strand breaks^61^. Consequently, an increased copy number of ORF-1 gDNA at 7 days might reflect LINE-1 integration into METH-damaged DNA. However, the majority of LINE-1 insertions are 5′–truncated and only 1 kbp in length^37^, suggesting that new LINE-1 insertions rarely contain ORF-1. In view of this fact, the increases in ORF-1 gDNA might reflect increased proliferation and/or survival of cells in neurogenic zones. Another potential interpretation of the observed results is that LINE-1 expression is induced without subsequent retrotransposition of the element, with the observed increases in ORF-1 gDNA being due to chromosome duplication, aneuploidy, or copy number variation^34^. The METH-induced increase in ORF-1 copy numbers within gDNA in the liver likely reflects neurotoxic effects of the drug on this tissue. METH induces oxidative damage to proteins, lipids and DNA, impairs mitochondria and reduces GSH supplies in the rodent liver to a similar extent to that in the rodent brain^62^. By contrast, there is no evidence for METH toxicity in animal or human muscle tissue (with the exception of the heart). In agreement with these data, there was no difference in the ORF-1 copy numbers in the muscle tissue of METH-exposed rats and saline controls. In summary, the observed increases in the ORF-1 gDNA levels in the dentate gyrus, as well as in the striatum and liver, very likely represent toxic METH-induced epigenetic events.

The decreases in ORF-1 mRNA levels observed in the dentate gyrus and striatum at 7 days after METH may reflect adaptive downregulation of LINE-1 transcription, ORF-1-containing cell loss within the neurogenic niches, decreased proliferation of these cells, and/or decreased survival of new neurons. Any of these events is plausible because high-dose METH decreases cell proliferation, differentiation, and survival, and induces the death of stem and progenitor cells in the SGZ and SVZ^60^,^63^. Alternatively, the decrease in ORF-1 mRNA might reflect, in part, the incorporation of LINE-1 into the genome without further ORF-1 mRNA production. The last scenario is supported by the positive correlation of the ORF-1 mRNA levels with the ORF-1 gDNA levels in the dentate gyrus; however, this result must be confirmed with larger group sizes.

The LINE-1 promoter region is strongly methylated at most CpG sites in the brain^37^,^64^. The methylation status of the LINE-1 promoter determines, in part, rat LINE-1 transcription^31^,^38^. We found only a small decrease (−1%) in the methylation levels of the LINE-1 promoter in the dentate gyrus, which was mainly due to the de-methylation of CpG9 and CpG3 sites. In the striatum, LINE-1 promoter hypomethylation, if it occurred, might have been diluted out, as the LINE-1 activation occured mainly in the SVZ. The required minimum degree of hypomethylation for LINE-1 gene activation is unknown. It is possible that, in our study, LINE-1 activation in the dentate gyrus was triggered by low-level de-methylation at the third and ninth individual CpG sites. This scenario is supported by the finding that the induction of LINE-1 transcription is dependent on the position rather than the number of hypomethylated CpGs^31^. In HeLa cells, methylation at the first seven CpGs in the LINE-1 promoter has been shown to be essential for LINE-1 inhibition^31^. The lack of correlation between the ORF-1 mRNA levels and the LINE-1 promoter methylation levels does not support this hypothesis. Our results together with the finding that morphine-increased LINE-1 expression does not correlate with LINE-1 hypomethylation^40^, point to mechanisms independent of cytosine methylation at the LINE-1 promoter CpG sites. The mechanism of transcriptional activation of repetitive elements has not been definitively elucidated; therefore, other factors may be involved in LINE-1 activation, such as SOX2, chromatin structure, DNA-editing proteins, the canonical WNT pathway, RNA helicases, small interfering RNAs^34^,^65^, small piRNAs^66^ and P1-LINE-1 RNA^67^, and HDACs^32^. Of these factors, HDACs are strong candidates for LINE-1 regulation after METH exposure^28^. In addition, the oxidation of methylated cytosines^68^, the hypomethylation of CpG sites other than the assessed CpG sites within the LINE-1 promoter region, or the methylation level of CpG sites outside the LINE-1 promoter region may also have played a part in activating of this element^69^. We focused on the LINE-1 promoter region only and did not distinguish between DNA methylation forms. Of note, LINE-1 sequences that are located within the LINE-1 promoter region do not share homology between species.

Both toxic and nontoxic regimens of METH alter the gene expression in striatal and hippocampal neurons^7^,^21^,^70^,^71^,^72^. Consequently, nontoxic METH doses might have effects on brain LINE-1 that are similar to those of neurotoxic METH doses. The METH-induced changes in gene expression are accompanied by changes in histone acetylation and deficits in certain HDACs^21^,^28^. On the other hand, histone acetylation and deficits in HDACs cause increased LINE-1 activity^32^, suggesting that METH-induced decreases in certain HDACs activate LINE-1, which in turn participates in the regulation of gene expression. The *in vivo* experiments did not determine whether neurotoxic METH doses induce LINE-1 retrotransposition and whether LINE-1 activity mediates METH neurotoxicity. If METH-mediated LINE-1 activation is followed by its retrotransposition *in vivo*, it might initiate a vicious cycle of neurotoxicity via DNA breakage^57^,^61^. Even in the absence of LINE-1 retrotransposition, LINE-1 might still participate in mediating of METH’s toxic effects *in vivo* via increased ORF-1 and ORF-2 expression because there is evidence of a physiological role of LINE-1 expression in responses to stress^73^,^74^ and in brain plasticity^33^,^37^. Altered levels of LINE-1 expression can trigger neurological impairments^34^, suggesting that LINE-1 induction may contribute to the development of cognitive impairments in human METH users.

## Methods

### Animals

Adult male Sprague-Dawley rats (Harlan, Indianapolis, IN, USA) (weighing 250–300 g on arrival) were pair-housed under a 12 h light/dark cycle in a temperature-controlled (20–22 °C) and humidity-controlled room. Food and water were available *ad libitum*. The animals were allowed to acclimatize for one week before the start of the study. All animal procedures were conducted between 7:00 A.M. and 7:00 P.M. in strict accordance with the National Institutes of Health (NIH) Guide for the Care and Use of Laboratory Animals and were approved by the Institutional Animal Care and Use Committee (IACUC) at Wayne State University (animal protocol #A 05-07-13). The description of animal procedures meets the ARRIVE recommended guidelines described by The National Centre for the Replacement, Refinement and Reduction of Animals in Research^75^.

### Administration of methamphetamine

(+)-Methamphetamine hydrochloride (METH, 10 mg/kg) (Sigma-Aldrich, St. Louis, MO) or saline (1 mL/kg) was administered to the rats every 2 h in four successive intraperitoneal (i.p.) injections. METH neurotoxicity is associated with hyperthermia, which peaks at approximately 1 h after each injection. Therefore, the core body temperatures of the rats were measured with a rectal probe digital thermometer (Thermalert TH-8; Physitemp Instruments, Clifton, NJ) before the beginning of the treatment (baseline temperatures) and at 1 h after each METH or saline injection. Rats were sacrificed by decapitation at 1 h (used for LINE-1 methylation analysis), 24 h or 7 days (used for multiple analysis) after the last injection of the drug or saline.

### Tissue collection

The brains were removed, dissected out into discrete brain areas (striatum, dentate gyrus, Ammon’s horn, prefrontal cortex, and cerebellum) and stored at −80 °C until assayed. The SVZ was dissected out together with the striatum whereas the SGZ was dissected out together with the dentate gyrus. The dentate gyrus was dissected out according to a previously described protocol^76^ modified for the rat brain. Briefly, the brain was cut sagittally to divide the hemispheres, which were then placed medial side up after removal of the regions posterior to lambda. The dentate gyrus and Ammon’s horn (CA1 and CA3) (which were visible upon removal of thalamus and hypothalamus) were dissected out using fine tip surgical instruments. Liver and muscle tissues were also collected and stored at –80°C until analysis.

### Real-time polymerase chain reaction and pyrosequencing

The levels of ORF-1 mRNA and ORF-1 gDNA in the dissected brain areas from METH-and saline-treated rats were determined using real-time quantitative polymerase chain reaction PCR (qPCR). DNA methylation of the first ten CpG sites within the LINE-1 promoter region was determined by pyrosequencing. GADPH was used as a reference gene in all analyses. The analyses were conducted at EpigenDx Inc. (Hopkinton, MA). DNA and RNA were extracted from two separated pieces of tissue. A 250 ng of total RNA from each tissue sample was used for cDNA synthesis with High Capacity cDNA Reverse Transcription kit (Life Technologies, Carlsbad, CA). The DNA and cDNA quantification was conducted by qPCR using primers complemented to sequences in LINE-1 ORF-1 region. The mRNA and gDNA copy numbers were calculated from the standard curves. For DNA methylation analysis, 500 ng of DNA from each rat brain tissue sample was first treated with bisulfite and subsequently purified using Zymo Research DNA columns (Zymo Research, Irvine, CA). A 1/20^th^ of eluted solution was used for each PCR. Biotinylated PCR products were bound to Streptavidin Sepharose HP (GE Healthcare, Waukesha, WI). Immobilized PCR products were purified using Pyrosequencing Vacuum Prep Tool (Qiagen, Valencia, CA) according to the manufacturer’s instructions. The 0.2 μM pyrosequencing primer was annealed to purified single-strained PCR product. The PCR products (10 uL) were sequenced using Pyrosequencing PSQ96 HS System (Biotage AB, Charlotte, NC). The methylation status of each locus was analyzed individually as a T/C SNP using QCpG software (Pyrosequencing, Qiagen). The data is expressed as LINE-1/GADPH ratios normalized to saline controls (mean ± SEM).

### Immunohistochemistry

Brain tissue from the rats sacrificed by decapitation at 24 h after the last injection of METH or saline was fixed in 4% paraformaldehyde for 24 h then incubated in 20% and 30% buffered glycerol concentrations for 24 h each (4 °C). Every other of the coronal sections (20 μm, 3–4/rat) from the SVZ (1.18–0.26 from Bregma) and the dentate gyrus (−3.12 to –4.68 from Bregma) were examined using immunofluorescence. Sections were pretreated with 1× citrate buffer for 40 min at 70 °C then allowed to cool to room temperature before being blocked in a blocking buffer (phosphate-buffered saline (PBS), 0.1% Triton X-100, and 5% bovine serum albumin (BSA)) for 1 h at room temperature. The sections were then incubated overnight at 4 °C with a chicken anti-ORF-2 (1:200, Rockland Immunochemicals Inc., Limerick, PA1) and a rabbit anti-doublecortin (1:100, Abcam, Cambridge, MA) primary antibodies. The next day, the sections were incubated for 3 h at room temperature with the corresponding secondary antibodies, anti-chicken Alexa-488 (1:1000) and anti-rabbit Alexa-594 (1:2000) (Life Technologies, Carlsbad, CA). The incubations with primary and secondary antibodies were separated by three 5 min-long washes with PBS that contained 0.1% Triton and 5% BSA. The nuclei were labeled with DRAQ5 dye (Life Technologies). The sections were mounted on slides using Flouromount mounting medium (Sigma-Adlrich). Images were captured using Leica TCS SPE-II confocal microscope under the 63× oil objective (Leica). The ORF-2 immunofluorescence was measured in four non-overlapping areas per slice and averaged. Mean of the averages was then calculated for each rat.

### Cell culture, cell transfection and drug treatments

PC12 cells (a rat adrenal gland pheochromocytoma cell line) and PA-1 cells (an ovarian teratocarcinoma cell line) (ATCC, Manassas, VA) were grown in Gibco RPMI-1640 medium (Life Technologies) containing 10% horse serum and 5% fetal bovine serum (FBS). The plasmids p99-LRE3-eGFP and JM111-eGFP (kindly gifted by Dr. Alysson Muotri, US San Diego, CA, US) were used for the transient transfection of PC12 and PA-1 cells and served as the LINE-1 retrotransposition indicator and negative control, respectively. The cells were grown to an approximately 80% confluence. Plasmid DNA (1.2 μg) was mixed with 2 μL Lipofectamine 2000 (Life Technologies) and added to the cells according to the manufacturer’s instructions. The cells were trypsinized two days after the transfection, re-seeded into a 6-well plate, and treated with 150 or 300 μM METH for 10–14 days. Puromycin (7.5 μg/mL) was added to the cells on the 1^st^ day of METH treatment. The cells were grown until the appearance of an eGFP fluorescence signal (10–14 days), which indicates LINE-1 retrotransposition^36^. The eGFP signal was recorded using the EVOS FL Cell Imaging System (Life Technologies). A reverse transcriptase inhibitor, azidothymidine (AZT, 20 μM, Sigma-Aldrich), was added to some wells to ascertain that the eGFP signal was the result of LINE-1 retrotransposition. The transfection efficiency of the cells was assessed using the positive control plasmid pCMV6-AC-GFP (OriGene, Inc., Rockville, MD) and was estimated to be 70–80%. Each experiment was conducted three times. To monitor ORF-2 expression, LRE3-eGFP-transfected PC12 cells were puromycin-selected, untreated or treated with 2 mM DA or 2 mM GLU and evaluated for viability on an hourly basis using propidium iodide. JM111-eGFP-tranfected cells served as controls. After 4 h, the cells were fixed in 4% paraformaldehyde and permeabilized with 0.1% Triton X-100 for 10 min. After 1 h blocking step with 5% BSA, the cells were incubated (overnight, 4 °C) with an anti-ORF-2 primary antibody (1:1000, Rockland Immunochemicals Inc., Limerick, PA), the corresponding anti-chicken Alexa-488 secondary antibody (1:400, 1 h at RT), and DAPI to visualize the nuclei. Images were recorded using EVOS FL microscope.

### Statistical analysis

The comparisons made in the study were pre-planned comparisons. We established *a priori* the SGZ and SVZ as potentially affected brain regions in METH-exposed rats and chose other samples based on existing knowledge regarding the METH and LINE-1 effects on the brain, liver and muscle. Differences between the control and METH groups were analyzed by the Student’s *t*-test and were followed by the Bonferroni correction (which adjusts for the probability of Type I errors in multiple comparisons). A two-way repeated-measures ANOVA followed by the Student-Newman-Keuls *post hoc* test was performed on the temperature data. Correlations between indices were determined using Pearson’s analysis. To avoid Type II errors (failures to reject a false null hypothesis), some data were grouped before analysis or are presented in two ways: (1) adjusted for Type I errors and (2) unadjusted. The results are expressed as the mean ± SEM. Significance was set at *p* < 0.05.

## Additional Information

**How to cite this article**: Moszczynska, A. *et al.* Neurotoxic Methamphetamine Doses Increase LINE-1 Expression in the Neurogenic Zones of the Adult Rat Brain. *Sci. Rep.* **5**, 14356; doi: 10.1038/srep14356 (2015).

## Figures and Tables

**Figure 1:**
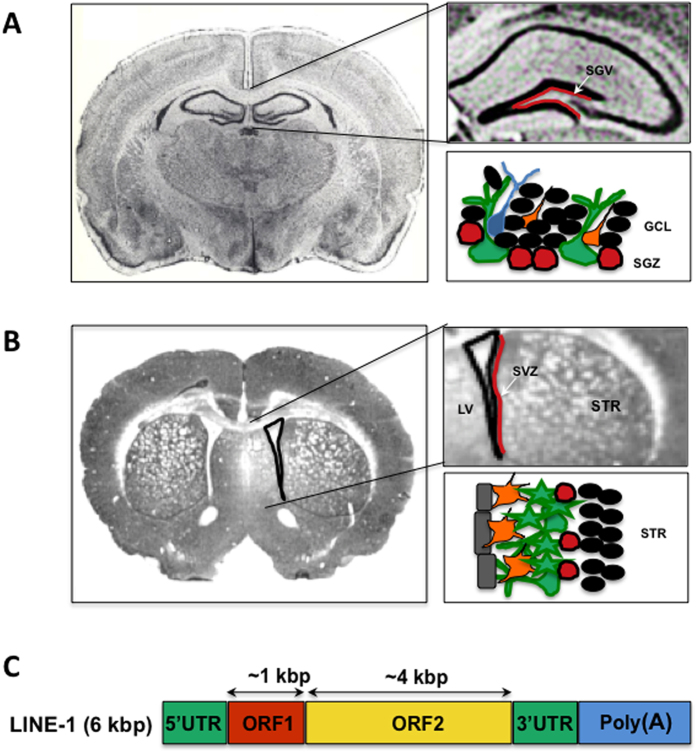
A schematic illustration of the composition of the subgranular zone (SGZ), subventricular zone (SVZ) and Long INterspersed Element 1 (LINE-1). In the adult rodent brain, (**A**) the SGZ lies below the granular cell layer of the dentate gyrus whereas (**B**) the SVZ lies between the lateral ventricle and the striatum. These regions share several components such as astroglial neural stem cells (green), neuroblasts (orange) and progenitor cells (red). Black circles represent mature granular cells and striatal cells by the SGZ and SVZ, respectively; blue denotes an immature neuron in the SGZ (based on^56^). (**C**) The LINE-1 element consists of the promoter-containing 5′untranslated region (5′UTR), 2 open reading frames (ORF-1 and ORF-2), and a 3′untranslated region (3′UTR) with a polyA tail (based on^34^). Abbreviations: GCL, the granular cell layer; kbp, kilobase pairs; LV, the lateral ventricle; STR, the striatum.

**Table 1:** The effect of binge methamphetamine (METH) on methylation status of LINE-1 promoter in several areas of rat brain assessed at 24 h after binge METH or saline (SAL).

Brain area	CpG1	CpG2	CpG3	CpG4	CpG5	CpG6	CpG7	CpG8	CpG9	CpG10	Average
*Striatum*											
SAL	84.5 ± 0.5	54.3 ± 0.3	73.6 ± 0.5	68.3 ± 0.7	77.8 ± 1.1	30.5 ± 0.1	99.7 ± 0.3	56.1 ± 0.7	65.7 ± 0.9	80.0 ± 0.3	69.0 ± 0.2
METH	85.3 ± 0.4	55.2 ± 0.3	74.0 ± 0.3	70.0 ± 0.8	77.4 ± 0.7	30.7 ± 0.3	100 ± 0	56.8 ± 0.7	65.7 ± 1.1	78.1 ± 0.4	69.3 ± 0.3
*Dentate gyrus*											
SAL	84.5 ± 0.4	54.9 ± 0.4	74.6 ± 0.2	69.9 ± 0.6	76.2 ± 0.6	30.9 ± 0.4	99.7 ± 0.3	57.4 ± 0.6	66.4 ± 0.2	78.2 ± 0.6	69.3 ± 0.2
METH	85.6 ± 0.4	54.9 ± 0.5	72.7 ± 0.3#	68.5 ± 0.8	76.8 ± 1.0	30.2 ± 0.2	99.4 ± 0.6	55.9 ± 0.2	62.9 ± 0.4#	79.2 ± 0.4	68.5 ± 0.3*
*Hippocampus*											
SAL	84.4 ± 0.2	54.5 ± 0.4	74.4 ± 0.2	70.1 ± 0.6	75.8 ± 0.8	30.9 ± 0.3	99.7 ± 0.2	57.5 ± 0.2	66.3 ± 1.0	80.1 ± 0.4	69.5 ± 0.1
METH	85.7 ± 0.4	54.8 ± 0.5	73.4 ± 0.4	69.5 ± 0.6	76.6 ± 0.6	30.6 ± 0.3	99.8 ± 0.2	56.0 ± 0.5	66.4 ± 0.4	78.4 ± 0.6	69.1 ± 0.2
*Frontal cortex*											
SAL	83.9 ± 0.3	54.5 ± 0.3	73.3 ± 0.4	68.8 ± 0.6	77.1 ± 0.6	29.6 ± 0.2	100 ± 0	54.6 ± 0.4	64.2 ± 0.6	78.1 ± 0.4	68.4 ± 0.2
METH	84.9 ± 0.4	53.9 ± 0.3	72.8 ± 0.4	69.2 ± 0.3	75.8 ± 0.5	29.7 ± 0.3	99.8 ± 0.2	54.8 ± 0.5	65.0 ± 0.5	76.9 ± 0.6	68.3 ± 0.2
*Cerebellum*											
SAL	86.0 ± 0.5	55.6 ± 0.3	74.8 ± 0.3	71.4 ± 0.8	77.0 ± 0.9	31.3 ± 0.3	100 ± 0	57.5 ± 0.3	66.5 ± 1.1	79.3 ± 0.7	69.9 ± 0.3
METH	86.5 ± 0.2	55.5 ± 0.4	75.9 ± 0.3	71.1 ± 0.3	77.0 ± 0.5	31.8 ± 0.3	99.9 ± 0.1	58.1 ± 0.5	67.4 ± 0.2	78.3 ± 0.3	70.1 ± 0.2

Statistically significant: *p < 0.05 (Student’s *t*-test), ^#^*p *< 0.005 (Student’s *t*-test with the Bonferroni correction), n = 5–7 rats/group.

First ten C-phosphate-G (CpG) sites within the promoter region were assessed for percent of methylation by pyrosequencing in saline- and METH-treated rats at 24 h after the last injection of the drug or saline. Average methylation of LINE-1 promoter was significantly decreased in the dentate gyrus (−1%) due to hypomethylation at the CpG3 and CpG9 site.

**Table 2:** The effect of binge methamphetamine (METH) on methylation status of LINE-1 promoter in the dentate gyrus and striatum assessed at 1 h after binge METH or saline (SAL).

Brain area	CpG1	CpG2	CpG3	CpG4	CpG5	CpG6	CpG7	CpG8	CpG9	CpG10	Average
*Striatum*											
SAL	86.1 ± 0.3	57.3 ± 0.5	66.0 ± 0.3	81.2 ± 0.4	98.6 ± 0.4	33.9 ± 0.4	100 ± 0	59.2 ± 0.4	66.6 ± 0.5	80.6 ± 0.5	72.8 ± 0.3
METH	86.6 ± 0.2	57.7 ± 0.1	66.4 ± 0.3	79.5 ± 0.4	97.4 ± 1.2	33.4 ± 0.3	100 ± 0	60.1 ± 0.5	67.4 ± 0.7	79.2 ± 0.7	73.0 ± 0.2
*Dentate gyrus*											
SAL	86.6 ± 0.3	57.1 ± 0.3	66.6 ± 0.8	80.3 ± 0.6	98.7 ± 0.9	32.6 ± 0.7	100 ± 0	58.5 ± 0.7	58.5 ± 0.7	66.2 ± 0.4	73.0 ± 0.2
METH	86.6 ± 0.2	58.0 ± 0.5	67.0 ± 0.8	81.0 ± 0.6	95.7 ± 0.6	34.5 ± 0.7	100 ± 0	60.2 ± 0.5	60.2 ± 0.5	67.2 ± 0.8	72.8 ± 0.4

First ten C-phosphate-G (CpG) sites within the promoter region were assessed for percent of methylation by pyrosequencing in saline- and METH-treated rats at 1 h after the last injection of the drug or saline. Average methylation of LINE-1 promoter was not significantly changed either in the dentate gyrus or striatum.
